# Association between prognostic nutritional index and long-term mortality in intensive care unit patients with pressure ulcers: A retrospective study

**DOI:** 10.1371/journal.pone.0341343

**Published:** 2026-02-10

**Authors:** Yamin Bo, Chao Song, Jinhui Zhang

**Affiliations:** 1 Department of Hematology, The Affiliated Hospital, Jiangsu University, Zhenjiang, Jiangsu, China; 2 Department of Emergency Medicine, The Affiliated Hospital, Jiangsu University, Zhenjiang, Jiangsu, China; 3 Department of Critical Care Medicine, The Affiliated Hospital, Jiangsu University, Zhenjiang, Jiangsu, China; Shuguang Hospital, CHINA

## Abstract

**Background:**

The prognostic nutritional index (PNI), an integrated marker of nutritional and immune status, has been proposed as a potential prognostic factor in critically ill patients. This study aimed to investigate the relationship between PNI and long-term mortality in intensive care unit (ICU) patients with pressure ulcers.

**Methods:**

Individuals with pressure ulcers were selected from the Medical Information Mart for Intensive Care IV (MIMIC-IV) database and divided into four categories according to the quartiles of the PNI. Several statistical methods were employed to evaluate the association between PNI and long-term mortality, including Kaplan-Meier survival analysis, multivariable Cox proportional hazards models, restricted cubic splines (RCS), receiver operating characteristic (ROC) curves, and subgroup analyses.

**Results:**

The median age of the 796 participants was 70.33 years (IQR: 60.91–79.96), with 451 (56.7%) being male. During the follow-up period, 476 patients (59.8%) died within 365 days, and 413 patients (51.9%) died within 180 days. Kaplan-Meier analysis indicated that patients with elevated PNI values had a significantly lower risk of mortality at both 365 and 180 days. After full adjustment for potential confounders, patients in the highest PNI quartile had significantly lower mortality risks compared to those in the lowest quartile (365-day mortality: HR = 0.598, 95% CI: 0.455–0.786, P < 0.001; 180-day mortality: HR = 0.558, 95% CI: 0.414–0.753, P < 0.001). A non-linear L-shaped relationship was identified between PNI and mortality, with threshold values of 28.45 for 365-day mortality and 29.15 for 180-day mortality.

**Conclusions:**

A lower PNI was significantly associated with increased risk of long-term mortality in ICU patients with pressure ulcers. These findings suggested the potential of PNI as a valuable prognostic biomarker in this population.

## Introduction

Pressure ulcers, also known as pressure injuries, refers to localized damage to the skin and/or underlying subcutaneous tissue, usually over a bony prominence, due to prolonged pressure or shear forces [1]. Patients admitted to intensive care units (ICUs) are commonly affected by pressure ulcers, which imposes both physical and emotional burdens and contribute to rising medical expenses and increased demands on nursing resources [2]. Multiple factors elevate the risk of pressure ulcers development in ICU patients, including hemodynamic instability, impaired consciousness, use of specific medications, malnutrition, and prolonged immobility [3]. Evidence indicates that the incidence of hospital-acquired pressure ulcers is nearly four times higher in ICU settings compared to general wards [4]. Furthermore, several studies have suggested that patients complicated with pressure ulcers may be associated with poorer clinical outcomes and increased mortality [5–7]. Therefore, the early identification of reliable prognostic predictors is critical for improving stratification, guiding interventions, and reducing mortality in this population.

The pathological mechanism of pressure ulcers is complex and not fully understood. Growing evidence indicates that the pathogenesis and progression of pressure ulcers are closely linked to immunological and nutritional status, and markers of these factors offering significant potential for improving preventive and therapeutic strategies of pressure ulcers [8–11]. Among these markers, serum albumin serves as an important clinical indicator to evaluate the nutritional status of individuals, which is predominantly synthesized by the liver, possesses anti-inflammatory and antioxidant properties [12]. Previous research has showed hypoalbuminemia as a significant factor impairing wound healing in patients with pressure ulcers [13]. Furthermore, lymphocytes, a type of immune cell in the body, are closely associated with the progression of inflammation and have been shown to play a role in coordinating the complex and dynamic inflammatory response in pressure ulcers [14]. The prognostic nutritional index (PNI), calculated by serum albumin and lymphocyte count, serves as an indicator of both nutritional and immune status [15]. It is widely recognized as a prognostic marker in cancer patients, where lower PNI values are consistently associated with higher mortality risk [16–18]. In recent years, PNI has been employed to assess the prognosis of individuals with various non-neoplastic conditions, including cardiovascular diseases, cerebrovascular diseases, autoimmune diseases, and infectious diseases, as well as postoperative complications [19–23]. Despite its growing prognostic utility across clinical settings, the role of PNI in predicting outcomes in patients with pressure ulcers has not been examined. Our primary hypothesis was that PNI values were significantly associated with mortality risk in critically ill patients with pressure ulcers, with lower values potentially indicating poorer outcomes. The objective of this study was to examine the relationship between the PNI and long-term all-cause mortality in individuals with pressure ulcers, with the goal of providing new insights into early prediction of clinical outcomes in this population.

## Methods

### Data source

All data were sourced from the MIMIC-IV database (version 3.1), a large publicly accessible database containing de-identified health information of patients admitted to ICU at Beth Israel Deaconess Medical Center (BIDMC) in Boston, Massachusetts, between 2008 and 2022. The dataset includes details on patient demographics, physiological parameters, clinical treatments, and mortality outcomes up to one year post-discharge. The corresponding author, Jinhui Zhang, completed the Collaborative Institutional Training Initiative (CITI) program and held a certification (Record ID: 65008026). Given that all patient datas have been de-identified, the Institutional Review Board (IRB) at BIDMC has waived the requirement for informed consent and approved the sharing of research resources. The study was conducted according to the Strengthening the Reporting of Observational Studies in Epidemiology (STROBE) reporting guideline. We began accessing the dataset for research on November 1, 2024.

### Study population

**Fig 1:**
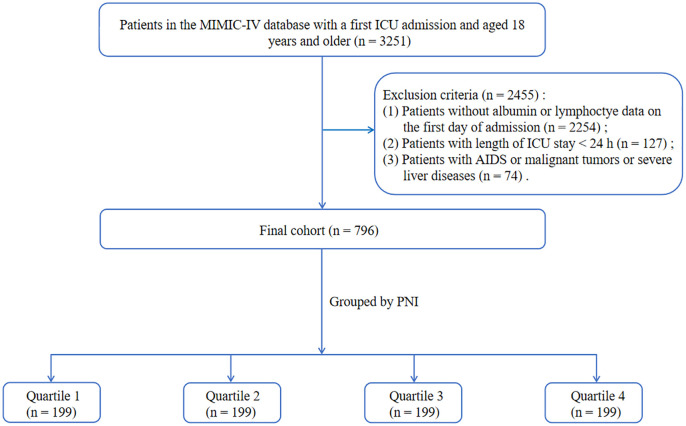
Flowchart of the study cohort.

The study cohort comprised patients diagnosed with pressure ulcers, defined according to International Classification of Diseases, Ninth Revision (ICD-9) and Tenth Revision (ICD-10). Patients were excluded based on the following criteria: (1) patients aged under 18 years; (2) patients with an ICU stay of less than 24 h; (3) patients who were not admitted to the ICU for the first time; (4) patients with lacking albumin or lymphocyte data on the first 24 h of ICU admission; (5) patients with a history of AIDS or malignant tumors or severe liver diseases defined by the ICD-9 and ICD-10. After applying these criteria, a total of 796 patients were included in the final study cohort. The patient selection process was illustrated in Fig 1.

### Data extraction

In this study, data extraction was performed using Navicat Premium 17 in conjunction with Structured Query Language (SQL), focusing on the following five domains:

Demographics: age, gender, weight, smoking, race;
Severity of illness scores: Sequential Organ Failure Assessment (SOFA) score, Oxford acute severity of illness score (OASIS);
Vital signs: temperature, heart rate, respiratory rate, systolic blood pressure (SBP), diastolic blood pressure (DBP), pulse blood oxygen saturation (SpO2);
Comorbidities and treatments: sepsis, myocardial infarction, heart failure, chronic pulmonary disease, cerebrovascular disease, hypertension, diabetes, renal failure, mechanical ventilation, renal replacement therapy;
5.Laboratory parameters: lymphocyte, platelet (PLT), hemoglobin, albumin, creatinine, blood urea nitrogen (BUN), prothrombin time (PT), glucose.

The PNI was calculated using the following formula: = [10 × serum albumin (g/dL) + 5 × lymphocyte count (10^9^/L)] [16]. Vital signs and all other variables were defined based on their initial recorded values within the first 24 hours of ICU admission. To minimize bias arising from sample exclusion, the proportion of missing values was assessed for each continuous variable. Variables with more than 20% missing values were excluded from the analysis. For variables with less than 20% missing data, missing values were imputed using a random forest-based multiple imputation method by the “mice” package of R software [24] (S1 Table).

### Outcomes

The primary endpoints of this study were defined as 365-day and 180-day all-cause mortality. Mortality data were retrieved from state or hospital death records. All individuals included in the MIMIC-IV database were followed for a minimum of one year.

### Statistical analysis

The PASS software was used to calculate the test’s effectiveness. A significance level (α) of 0.05 was set, with a total sample size of 796, resulting in a power of 95.6% to analyze the relationship between PNI and patient outcomes. Continuous variables were expressed as mean ± standard deviation (SD) or median (interquartile range, IQR), and were compared using the Student’s t-test or Mann-Whitney U test, as appropriate. Categorical variables were presented as frequencies (percentages) and were compared using the chi-square test or Fisher’s exact test. Kaplan-Meier analysis was performed to assess the incidence of primary outcomes, with stratification based on PNI levels. To mitigate potential multicollinearity, variables with a variance inflation factor (VIF) exceeding 5 were excluded from the model. Clinically relevant variables and those significantly associated with the prognosis were then included in the multivariate regression model: model 1: unadjusted; model 2: adjusted for age, gender, and race; model 3: adjusted for age, gender, race, weight, smoking, temperature, SBP, DBP, SpO2, sepsis, myocardial infarction, heart failure, chronic pulmonary disease, cerebrovascular disease, hypertension, diabetes, renal failure, and renal replacement therapy. In all models, the lowest quartile of the PNI was used as the reference group. The proportional hazards (PH) assumption for the Cox models was assessed using the Schoenfeld residuals tests. In the restricted cubic splines (RCS) model, we also adjusted for confounding factors: age, gender, race, weight, smoking, temperature, SBP, DBP, SpO2, sepsis, myocardial infarction, heart failure, chronic pulmonary disease, cerebrovascular disease, hypertension, diabetes, renal failure, and renal replacement therapy. If a nonlinear relationship was observed, the threshold inflection point was identified using the highest likelihood. The ability of the PNI to predict all-cause mortality was evaluated using receiver operating characteristic (ROC) curve analysis, with the area under the curve (AUC) quantifying predictive performance. Model calibration was assessed via the Hosmer-Lemeshow test. Additionally, stratified analyses were conducted to examine the consistency of PNI’s prognostic effect across subgroups, including age, gender, sepsis, hypertension, diabetes, myocardial infarction, heart failure, chronic pulmonary disease, and cerebrovascular disease. All statistical analyses were performed using SPSS version 26.0 and R language version 4.0.4. A two-sided P-value < 0.05 was considered statistically significant.

## Results

This study enrolled 796 patients with pressure ulcers. The study population had a median age of 70.33 years (IQR: 60.91–79.96), and the proportion of males was 56.7%. The median PNI was 32.09 (IQR: 27.41–37.27). Mortality rates at 180 and 365 days were 51.9% and 59.8%, respectively.

### Baseline characteristics of study individuals

Table 1 showed the baseline characteristics of the patient cohort, stratified according to PNI quartiles. The cohort was divided into four groups based on admission PNI values: Q1 (PNI ≤ 27.40), Q2 (27.40 < PNI ≤ 32.08), Q3 (32.08 < PNI ≤ 37.26), and Q4 (PNI > 37.26). The median PNI values for these groups were 24.80 (IQR: 22.00–26.27), 29.74 (IQR: 28.55–30.95), 34.65 (IQR: 33.45–35.75), and 41.33 (IQR: 38.98–45.20), respectively. Patients in the highest PNI group exhibited higher values for weight, temperature, SBP, DBP, lymphocyte, PLT, hemoglobin, and albumin, alongside lower SOFA scores and PT levels. Additionally, this group had a higher incidence of cerebrovascular disease, while the prevalence of sepsis and the need for renal replacement therapy were lower compared to patients in the lower PNI groups. As PNI levels increased, there was a gradual reduction in both 360-day mortality (69.3% vs. 61.3% vs. 57.3% vs. 51.3%, P = 0.003) and 180-day mortality (62.3% vs. 53.3% vs. 51.8% vs. 40.2%, P < 0.001).

**Table 1:** Baseline characteristics according to PNI quartiles.

Characteristics	Overall n = 796	Quartile 1 PNI ≤ 27.40 n = 199	Quartile 2 27.40 < PNI ≤ 32.08 n = 199	Quartile 3 32.08 < PNI ≤ 37.26 n = 199	Quartile 4 PNI > 37.26 n = 199	P
**Age, years**	70.33 (60.91-79.96)	70.01 (61.60-80.33)	70.32 (61.10-81.07)	70.24 (60.41-80.05)	70.81 (58.94-79.61)	0.947
**Male, n (%)**	451 (56.7)	105 (52.8)	115 (57.8)	114 (57.3)	117 (58.8)	0.629
**Weight, Kg**	73.5 (60.9-90.9)	69.1 (58.0-83.4)	74.0 (60.2-90.2)	75.8 (63.3-93.7)	76.5 (63.8-94.4)	0.038
**Smoking, n (%)**	40 (5.0)	9 (4.5)	9 (4.5)	12 (6.0)	10 (5.0)	0.889
**Race, n (%)**						0.067
White	509 (63.9)	130 (65.3)	135 (67.8)	129 (64.8)	115 (57.8)	
Black	108 (13.6)	19 (9.5)	24 (12.1)	28 (14.1)	37 (18.6)	
Asian	20 (2.5)	10 (5.0)	4 (2.0)	2 (1.0)	4 (2.0)	
Others	159 (20.0)	40 (20.1)	36 (18.1)	40 (20.1)	43 (21.6)	
**Severity of Illness**						
SOFA	7 (4-10)	7 (5-10)	7 (3-10)	7 (4-10)	6 (4-9)	0.043
OASIS	35 (29-42)	36 (31-43)	36 (29-41)	34 (29-42)	34 (29-42)	0.143
**Vital Signs**						
Temperature, °C	36.72 (36.39-37.11)	36.58 (36.22-37.00)	36.72 (36.39-37.17)	36.72 (36.39-37.09)	36.78 (36.44-37.11)	0.048
Heart rate, bpm	94 (80-110)	98 (80-113)	96 (80-107)	95 (82-110)	90 (76-108)	0.152
Respiratory rate, bpm	20 (16-25)	20 (16-25)	19 (16-23)	21 (18-26)	20 (16-24)	0.046
SBP, mmHg	114 (100-132)	110 (97-126)	116 (101-130)	114 (100-133)	121 (103-140)	0.001
DBP, mmHg	64 (53-76)	60 (47-72)	64 (54-76)	63 (53-75)	67 (46-80)	< 0.001
SpO2, %	98 (95-100)	97 (94-100)	98 (95-100)	97 (94-100)	98 (96-100)	0.020
**Commorbidities, n (%)**						
Sepsis	611 (76.8)	162 (81.4)	163 (81.9)	150 (75.4)	136 (68.3)	0.004
Myocardial infarct	154 (19.3)	37 (18.6)	37 (18.6)	45 (22.6)	35 (17.6)	0.593
Heart failure	324 (40.7)	71 (35.7)	82 (41.2)	85 (42.7)	86 (43.2)	0.398
Chronic pulmonary disease	206 (25.9)	47 (23.6)	50 (25.1)	48 (24.1)	61 (30.7)	0.351
Cerebrovascular disease	127 (16.0)	24 (12.1)	33 (16.6)	27 (13.6)	43 (21.6)	0.048
Hypertension	228 (28.6)	52 (26.1)	48 (24.1)	60 (30.2)	68 (34.2)	0.122
Diabetes	341 (42.8)	71 (35.7)	86 (43.2)	91 (45.7)	93 (46.7)	0.107
Renal failure	306 (38.4)	81 (40.7)	75 (37.7)	75 (37.7)	75 (37.7)	0.903
**Treatments, n (%)**						
Mechanical ventilation	383 (48.1)	99 (49.7)	93 (46.7)	91 (45.7)	100 (50.3)	0.757
Renal replacement therapy	166 (14.6)	50 (25.1)	38 (19.1)	49 (24.6)	29 (14.6)	0.029
**Laboratory parameters**						
Lymphocyte, x 10^9/L	0.94 (0.54-1.41)	0.56 (0.34-0.87)	0.81 (0.47-1.16)	1.01 (0.70-1.37)	1.54 (0.99-2.26)	< 0.001
PLT, x 10^9/L	216 (132-305)	186 (102-289)	221 (131-309)	200 (132-298)	242 (166-326)	0.001
Hemoglobin, g/dL	9.5 (8.0-11.0)	8.6 (7.5-10.0)	9.1 (7.8-10.4)	10.0 (8.7-11.3)	10.3 (8.8-12.1)	< 0.001
Albumin, g/dL	2.7 (2.3-3.1)	2.1 (1.8-2.3)	2.6 (2.4-2.8)	2.9 (2.7-3.1)	3.4 (3.1-3.7)	< 0.001
Creatinine, mg/dL	1.4 (0.8-2.6)	1.2 (0.7-2.4)	1.4 (0.9-2.6)	1.5 (0.8-2.8)	1.3 (0.7-2.3)	0.170
BUN, mg/dL	34 (21-56)	31 (18-52)	37 (22-62)	35 (22-60)	31 (20-53)	0.076
PT, s	15.3 (13.4-19.5)	15.7 (13.8-19.4)	15.6 (13.6-18.9)	15.8 (13.4-21.2)	14.3 (12.9-18.0)	0.001
Glucose, mg/dL	129 (101-172)	121 (95-168)	124 (101-162)	139 (104-197)	132 (103-180)	0.031
PNI	32.09 (27.41-37.27)	24.80 (22.00-26.27)	29.74 (28.55-30.95)	34.65 (33.45-35.75)	41.33 (38.98-45.20)	< 0.001
**Outcome**						
LOS hospital, days	14.7 (7.6-27.2)	15.1 (7.0-25.8)	13.9 (8.1-27.9)	14.7 (8.8-28.6)	14.6 (6.5-27.2)	0.875
180-day mortality, n (%)	413 (51.9)	124 (62.3)	106 (53.3)	103 (51.8)	80 (40.2)	< 0.001
365-day mortality, n (%)	476 (59.8)	138 (69.3)	122 (61.3)	114 (57.3)	102 (51.3)	0.003

Abbreviations: PNI, prognostic nutritional index; SOFA, Sequential organ failure assessment score; OASIS, Oxford acute severity of illness score; SBP, systolic blood pressure; DBP, diastolic blood pressure; SpO2, pulse blood oxygen saturation; PLT, platelets; BUN, blood urea nitrogen; PT, prothrombin time; LOS, length of stay.

Table 2 compared the baseline characteristics between survivors and non-survivors during the 365-day follow-up. Non-survivors tended to be older and exhibited higher rates of sepsis, heart failure, chronic pulmonary disease, renal failure, and renal replacement therapy. They also showed higher SOFA and OASIS scores, respiratory rate, creatinine, BUN, and PT levels, while weight, temperature, lymphocyte, PLT, hemoglobin, albumin, and PNI were significantly lower than in survivors. For validity of the results, we also reported the baseline characteristics and outcomes of the excluded and included patients. More detailed results were presented in S2 Table.

**Table 2:** Baseline characteristics of the survivors and non-survivors groups.

Characteristics	Overall n = 796	Survivors n = 320	Non-survivors n = 476	P
**Age, years**	70.33 (60.91-79.96)	66.61 (55.90-75.37)	73.24 (64.49-81.90)	< 0.001
**Male, n (%)**	451 (56.7)	176 (55.0)	275 (57.8)	0.439
**Weight, Kg**	73.5 (60.9-90.9)	76.9 (63.7-94.5)	71.6 (59.1-87.8)	0.011
**Smoking, n (%)**	40 (5.0)	16 (5.0)	24 (5.0)	0.979
**Race, n (%)**				0.357
White	509 (63.9)	195 (60.9)	314 (66.0)	
Black	108 (13.6)	45 (14.1)	63 (13.2)	
Asian	20 (2.5)	7 (2.2)	13 (2.7)	
Others	159 (20.0)	73 (22.8)	86 (18.1)	
**Severity of Illness**				
SOFA	7 (4-10)	6 (3-9)	7 (5-10)	< 0.001
OASIS	35 (29-42)	33 (27-40)	37 (30-43)	< 0.001
**Vital Signs**				
Temperature, °C	36.72 (36.39-37.11)	36.83 (36.44-37.17)	36.61 (36.33-37.00)	< 0.001
Heart rate, bpm	94 (80-110)	94 (80-107)	95 (80-111)	0.307
Respiratory rate, bpm	20 (16-25)	19 (16-23)	20 (17-26)	0.005
SBP, mmHg	114 (100-132)	117 (100-134)	113 (99-131)	0.100
DBP, mmHg	64 (53-76)	64 (55-76)	63 (52-77)	0.284
SpO2, %	98 (95-100)	98 (95-100)	98 (95-100)	0.885
**Commorbidities, n (%)**				
Sepsis	611 (76.8)	226 (70.6)	385 (80.9)	0.001
Myocardial infarct	154 (19.3)	52 (16.3)	102 (21.4)	0.070
Heart failure	324 (40.7)	110 (34.4)	214 (45.0)	0.003
Chronic pulmonary disease	206 (25.9)	67 (20.9)	139 (29.2)	0.009
Cerebrovascular disease	127 (16.0)	43 (13.4)	84 (17.6)	0.112
Hypertension	228 (28.6)	111 (34.7)	117 (24.6)	0.002
Diabetes	341 (42.8)	126 (39.4)	215 (45.2)	0.105
Renal failure	306 (38.4)	87 (27.2)	219 (46.0)	< 0.001
**Treatments, n (%)**				
Mechanical ventilation	383 (48.1)	151 (47.2)	232 (48.7)	0.667
Renal replacement therapy	166 (14.6)	52 (16.3)	114 (23.9)	0.009
**Laboratory parameters**				
Lymphocyte, x 10^9/L	0.94 (0.54-1.41)	1.00 (0.60-1.45)	0.87 (0.51-1.40)	0.068
PLT, x 10^9/L	216 (132-305)	232 (142-318)	208 (124-302)	0.045
Hemoglobin, g/dL	9.5 (8.0-11.0)	9.8 (8.2-11.3)	9.3 (7.9-10.8)	0.004
Albumin, g/dL	2.7 (2.3-3.1)	2.9 (2.4-3.2)	2.6 (2.2-3.0)	< 0.001
Creatinine, mg/dL	1.4 (0.8-2.6)	1.2 (0.7-1.9)	1.5 (0.9-2.8)	< 0.001
BUN, mg/dL	34 (21-56)	28 (18-46)	39 (23-62)	< 0.001
PT, s	15.3 (13.4-19.5)	14.7 (13.2-18.6)	15.6 (13.6-19.8)	0.016
Glucose, mg/dL	129 (101-172)	125 (100-167)	131 (101-174)	0.421
PNI	32.09 (27.41-37.27)	33.85 (28.58-38.33)	31.28 (26.71-36.37)	< 0.001

Abbreviations: PNI, prognostic nutritional index; SOFA, Sequential organ failure assessment score; OASIS, Oxford acute severity of illness score; SBP, systolic blood pressure; DBP, diastolic blood pressure; SpO2, pulse blood oxygen saturation; PLT, platelets; BUN, blood urea nitrogen; PT, prothrombin time.

### PNI and clinical outcomes

**Fig 2:**
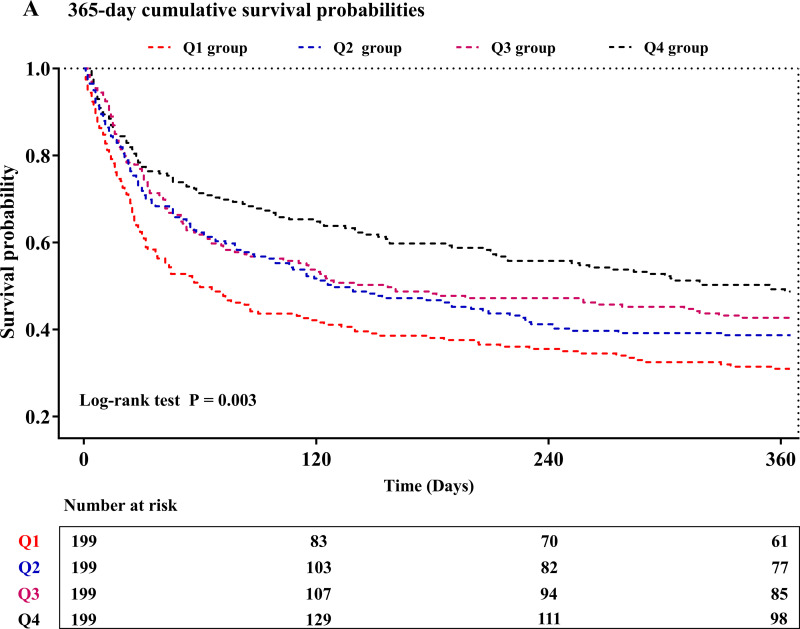
Kaplan-Meier curves of PNI for 365-day (A) and 180-day (B) mortality.

We employed Kaplan-Meier survival analysis stratified by PNI quartiles to compare the primary outcomes between groups. The results indicated that patients in lowest quartile exhibited a significantly elevated risk of both 365-day (log-rank P = 0.003) and 180-day mortality (log-rank P < 0.001), as shown in Figs 2A and 2B. Univariate Cox regression results were presented in S3 Table. Variance inflation factors, detailed in S4 Table, confirmed no multicollinearity among the variables. To further explore the association between PNI and mortality, we employed Cox proportional hazards regression models (Table 3). The Schoenfeld residual tests indicated no evidence of violation of the PH assumption for the core variable PNI (all P > 0.05) (S5 Table). Patients in higher PNI quartiles were significantly associated with a reduced risk of 365-day mortality across all models: unadjusted [HR, 0.568 (95% CI 0.440–0.734); P < 0.001], partially adjusted [HR, 0.547 (95% CI 0.423–0.707); P < 0.001], and fully adjusted [HR, 0.598 (95% CI 0.455–0.786); P < 0.001]. A significant trend toward decreased risk with increasing PNI quartile was observed (P for trend < 0.001). Consistent results were found in the multivariate analysis of 180-day mortality. Furthermore, we stratified the study population by gender and found that in both males and females, a significant association between PNI and all-cause mortality was observed (S6 Table).

**Table 3:** Cox proportional hazard models for 365-day and 180-day mortality.

Variables	Model 1			Model 2			Model 3		
	HR (95% CI)	p-value	P for trend	HR (95% CI)	p-value	P for trend	HR (95% CI)	p-value	P for trend
**365-day mortality**									
PNI (Quartiless)			< 0.001			< 0.001			< 0.001
Quartile 1 group	ref			ref			ref		
Quartile 2 group	0.764 (0.599-0.975)	0.030		0.715 (0.560-0.913)	0.007		0.762 (0.595-0.976)	0.032	
Quartile 3 group	0.691 (0.539-0.886)	0.004		0.659 (0.514-0.845)	0.001		0.681 (0.528-0.880)	0.003	
Quartile 4 group	0.568 (0.440-0.734)	< 0.001		0.547 (0.423-0.707)	< 0.001		0.598 (0.455-0.786)	< 0.001	
**180-day mortality**									
PNI (Quartiless)			< 0.001			< 0..001			< 0.001
Quartile 1 group	ref			ref			ref		
Quartile 2 group	0.743 (0.573-0.963)	0.025		0.700 (0.539-0.908)	0.007		0.747 (0.574-0.972)	0.030	
Quartile 3 group	0.707 (0.544-0.918)	0.009		0.676 (0.521-0.879)	0.003		0.700 (0.534-0.916)	0.009	
Quartile 4 group	0.512 (0.386-0.678)	< 0.001		0.494 (0.373-0.656)	< 0.001		0.558 (0.414-0.753)	< 0.001	

Model 1 was unadjusted.

Model 2 was adjusted for age, gender, and race.

Model 3 was adjusted for the variables in model 2 and further adjusted for weight, smoking, temperature, SBP, DBP, SpO2, sepsis, myocardial infarction, heart failure, chronic pulmonary disease, cerebrovascular disease, hypertension, diabetes, renal failure, and renal replacement therapy.

**Fig 3:**
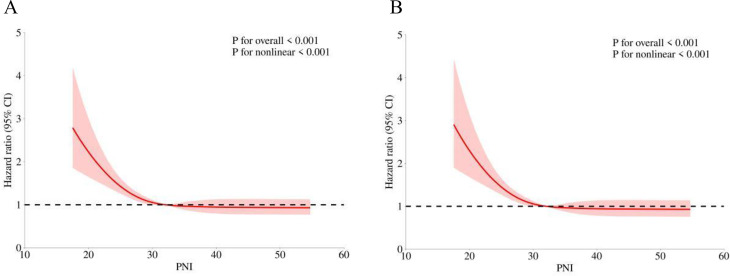
RCS of PNI with 365-day (A) and 180-day (B) mortality.

**Fig 4:**
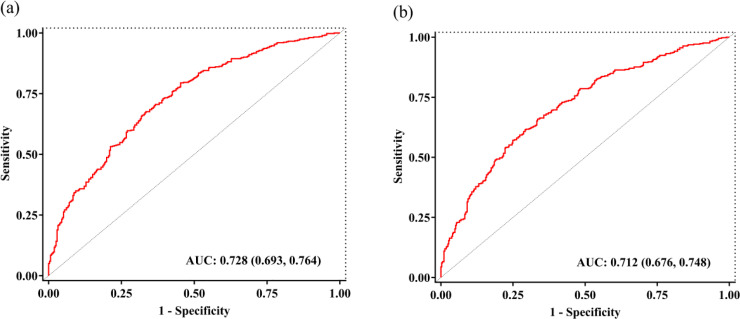
ROC curve for 365-day (a) and 180-day (b) mortality prediction model.

To evaluate potential non-linear relationship between the PNI and mortality, we utilized RCS models. As shown in Figs 3A and 3B, an L-shaped association was identified between PNI and both 365-day and 180-day mortality in the fully adjusted model. This non-linearity was further examined using both the standard Cox proportional hazards model and a two-piecewise Cox proportional hazards model, with log-likelihood ratio tests for both models showing statistically significant results (P < 0.05). Threshold effect analysis (Table 4) revealed inflection points at PNI values of 28.45 for 365-day mortality and 29.15 for 180-day mortality. Below these thresholds, each unit increase in PNI was significantly associated with lower mortality risk: HR 0.922 (95% CI:0.876–0.970; P = 0.002) for 365-day mortality, and HR 0.920 (95% CI: 0.877–0.965; P < 0.001) for 180-day mortality. The predictive model demonstrated moderate discrimination for both 365-day and 180-day mortality, with AUC values of 0.728 (95% CI: 0.693–0.764) and 0.712 (95% CI: 0.676–0.7748), respectively, as determined by ROC analysis (Figs 4a and 4b). Additionally, pseudo-R² values of 0.232 for 365-day and 0.196 for 180-day mortality suggested adequate model fit.

**Table 4:** Threshold effect analysis of PNI on 365-day and 180-day mortality.

Outcome	HR (95%CI)	P
**365-day mortality**		
Model 1 Fitting model by standard linear regression	0.985 (0.972-0.999)	0.031
Model 2 Fitting model by two-piecewise linear regression		
Inflection point	28.45	
< 28.45	0.922 (0.876-0.970)	0.002
≥ 28.45	0.999 (0.996-1.002)	0.442
P for likelihood test		< 0.001
**180-day mortality**		
Model 1 Fitting model by standard linear regression	0.985 (0.971−0.999)	0.038
Model 2 Fitting model by two-piecewise linear regression		
Inflection point	29.15	
< 29.15	0.920 (0.877-0.965)	< 0.001
≥ 29.15	0.999 (0.996-1.002)	0.503
P for likelihood test		< 0.001

### Stratified analyses

In addition, we conducted subgroup analyses of patient outcomes based on factors such as age, gender, sepsis, hypertension, diabetes, myocardial infarction, heart failure, chronic pulmonary disease, and cerebrovascular disease. The association between PNI and 365-day mortality risk remained consistent across most subgroups, including all gender, sepsis, hypertension, diabetes, heart failure, and chronic pulmonary disease subgroups as well as in individuals aged > 65 years, without myocardial infarct, or without cerebrovascular disease. Additionally, no significant interactions between the factors in the subgroup examination and the PNI were found (all p-values for interaction > 0.05) (S7 Table). Similar results were obtained in stratified analyses of the PNI and 180-day mortality (S8 Table).

## Discussion

In the present study, we assessed the association between the PNI and prognosis in critically ill patients with pressure ulcers from a United States (US) cohort. We discovered the L-shaped relationships between PNI levels and 365-day and 180-day all-cause mortality in the study, indicating that there was a significant association between lower PNI levels and higher risk of mortality among critically ill patients with pressure ulcers. Thus, the PNI serves as a promising clinical decision-making tool for clinicians and may be an independent risk factor for mortality in critically ill patients with pressure ulcers.

The underlying pathological mechanisms of pressure ulcers remain complex and have not yet been fully elucidated. Given the robust evidence indicating that the development and progression of pressure ulcers are closely linked to nutritional status and immunity [25,26]. Malnutrition serves as a critical risk factor in the onset of pressure ulcers and the delayed wound healing [27]. Furthermore, prolonged nutritional deficiency also diminishes neovascularization and phagocytosis, extends the inflammatory phase, and weakens the biomechanical strength of repaired tissue [28]. According to studies by Verbrugghe et al. and Iizaka et al., malnutrition led to prolonged hospital stays, higher infection risk, and increased mortality in patients with pressure ulcers, along with directly impacting disease progression [29,30]. Levine et al. demonstrated that nutritional optimization could promote the healing of pressure injuries [31]. The immune response plays a critical role in the healing of pressure ulcers by activating specific immune cells and mediators to initiate inflammation, facilitate wound debridement, and promote tissue repair [32]. Immunocompromised individuals, including patients with diabetes or those critically ill in the ICU, are at elevated risk for chronic pressure ulcers due to impaired immune responses and delayed wound healing [33]. Therefore, immune and nutritional factors play an important role in the pathogenesis of pressure ulcers, and the management of pressure ulcers should take these factors into account.

Albumin serves as the predominant protein in plasma, with its essential roles encompassing the maintaining colloid osmotic pressure, providing antioxidant and anticoagulant effects, modulating immune responses, and preserving vascular integrity [34]. Protein deficiency disrupts immune cell function and diminishes both fibroblast activity and collagen synthesis, thereby impairing collagen deposition and hindering wound healing [28]. Flattau et al. identified that serum albumin level was a strong marker of 90‐day and 180‐day mortality in adult hospitalised patients with pressure ulcers [35]. In patients with pressure ulcers undergoing flap reconstruction, serum albumin levels ≥ 2.5 g/dL were associated with a lower incidence of postoperative complications and a greater probability of complete wound healing [36]. Similarly, lymphocyte count serves as a key marker of cellular immunity and a decrease in lymphocytes may lead to immunosuppression, thereby elevating the development of pressure ulcers and was associated with a higher risk of mortality [37,38]. Moreover, immune dysregulation during the wound healing process can result in sustained inflammation and hinder timely wound healing [32]. Given the complexity of pressure ulcers’s clinical course, which is influenced by multiple factors, relying on a single predictive indicator is often insufficient for accurately forecasting patient outcomes. Calculated from serum albumin and lymphocyte counts, the PNI serves as an integrative biomarker that reflects both nutritional and immune status [39], significantly improving prognostic evaluation for patients with pressure ulcers.

Several previous studies have explored the prognostic value of the PNI in relation to mortality and adverse clinical outcomes across various patient populations. For instance, Xu et al. identified a correlation between elevated PNI levels and improved disease-free survival in patients with breast cancer [40]. In the study by Li et al., a higher pretreatment PNI was served as a favorable prognostic indicator among patients with prostate cancer undergoing androgen deprivation therapy [41]. Zhang et al. demonstrated that in patients with type 2 diabetes, the PNI was not only associated with the onset of diabetic kidney disease but also independently predicted all-cause mortality [42]. Wang et al. documented a significant inverse relationship between the PNI and all-cause mortality in patients with community-acquired pneumonia [43], while another study by Wei et al. identified that reduced PNI was correlated with poorer outcomes in patients with severe COVID-19 [44]. Moreover, Wu et al. suggested that low PNI was an independent risk factor for elevated mortality in sepsis patients [45]. In the context of cerebrovascular diseases, PNI has also been recognized as a predictor of long-term mortality among older adults with ischemic stroke [20]. Huang et al. showed that low PNI independently predicted one-year all-cause mortality in acute myocardial infarction patients admitted to the ICU [46], while Chang et al. found an association between reduced PNI and elevated long-term mortality risk in patients undergoing coronary interventions for acute coronary syndrome [47]. Additionally, our study revealed that the lower PNI was more strongly associated with long-term all-cause mortality in severe patients with pressure ulcers, which is consistent with Wang et al.’s findings that a reduced PNI increased mortality risk in rheumatoid arthritis patients [48], indicating that the PNI has significant predictive value for all-cause mortality across various critically ill populations, including patients with pressure ulcers.

Our study also revealed an L-shaped relationship between PNI and both 180- and 365-day all-cause mortality, which indicated that the protective effect of PNI had a certain threshold. The turning point was around the median level of PNI, which was consistent with observations from prior research on the association of PNI with other diseases, including cardiovascular disease and chronic obstructive pulmonary disease [49,50]. Specifically, a lower PNI was associated with an increased risk of all-cause mortality in pressure ulcer patients only when PNI levels fell below the turning point. There are several potential mechanisms underlying the association between PNI and mortality in patients with pressure ulcers. Malnutrition, reflected by a low PNI, can impair mitochondrial function, promoting the production of reactive oxygen species and activating inflammatory cascades, consequently increasing susceptibility to severe infections [51,29]. Moreover, the relationship between inflammation and nutritional status is bidirectional, with inflammation potentially leading to malnutrition through inducing insulin resistance and stimulating catabolic processes [51]. Malnutrition exacerbates chronic inflammation and induces immunosuppression, thereby diminishing the host’s capacity to fight infections and adversely affecting the healing of pressure ulcers [52]. In addition, low PNI has been associated with a higher prevalence of comorbidities, including pneumonia and renal dysfunction, which independently increase the risk of mortality [53,54]. Collectively, these physiological alterations may contribute to the development and advancement of pressure ulcers, ultimately leading to unfavorable clinical prognoses.

In addition, subgroup analysis validated the lower PNI as a stable predictor of both 180-day and 365-day mortality in patients with pressure ulcers, with its prognostic utility holding consistent across diverse demographic and clinical conditions. Our subgroup analysis demonstrated that the value of the PNI in predicting all-cause mortality was consistent in male and female patients. However, we did not find any significant association between the PNI and all-cause mortality in patients with myocardial infarction or cerebrovascular disease at baseline. The reason for this phenomenon may be that patients diagnosed with these comorbidities were more likely to have received appropriate treatment or adopted healthier lifestyle habits. Another important finding of the present study was that the association between PNI and 365-day mortality seemed to be more significant in older patients, which was consistent with the previous study [55]. A potential explanation for this finding was that aging was often associated with multiple comorbidities, sarcopenia, reduced nutrient intake, and immunosenescence, all of which may possess a greater tolerance for PNI fluctuations during the initial acute response upon ICU admission [56–58]. Previous studies have reported inconsistent findings regarding the impact of PNI on prognosis across distinct age cohorts. Liu et al. identified a significant association between low PNI and 28-day mortality in patients with chronic obstructive pulmonary disease, irrespective of patient age [50]. In an age-stratified analysis of hemodialysis patients, Miyasato et al. found that the lower PNI was more predictive of mortality in the subgroup under 65 years than in older patients [59]. Wang et al. demonstrated a more pronounced association between reduced PNI and 90-day mortality in patients under 65 years compared to older patients with community-acquired pneumonia [43]. This discrepancy may be attributed to the lower incidence of in-hospital mortality among patients under 65 years in their study, which could have resulted in insufficient statistical power.

To the best of our knowledge, this study represented the first investigation of the relationship between the PNI and all-cause mortality in critically ill patients with pressure ulcers. However, several limitations should be acknowledged. First, as a retrospective analysis of observational data from the single-center MIMIC-IV database, this study inherently limits the establishment of causal relationships. Although we adjusted for numerous covariates and performed subgroup analyses, the potential influence of confounding factors on our findings cannot be completely eliminated. Second, a number of patients were excluded due to incomplete data on albumin or lymphocyte levels, which could potentially introduce selection bias into the study. Third, data on nutritional support interventions were unavailable, limiting our understanding of how these treatments might interact with PNI and influence mortality risk. Fourth, the study’s sample size is moderate, necessitating further cohort studies with larger sample sizes to substantiate our conclusions. Fifth, we did not compare the prediction power of PNI for mortality with that of more complex nutritional indicators, including malnutrition-inflammation score, because the specific scoring data could not be obtained from the database. The potential impact of this on the conclusions drawn from our study necessitates further investigation. Sixth, as the data for this study were sourced from the MIMIC-IV database, which represented only the US population, potentially limiting its generalizability. Future studies are necessary to validate these findings across different ethnic populations. Finally, as the PNI were measured only at a single baseline time point, we could not evaluate how their dynamic changes during hospitalization might affect patient outcomes. Future research should incorporate serial measurements to more accurately delineate how dynamic changes in these metabolic indices affect prognosis after pressure ulcers.

## Conclusions

In conclusion, our study identified that the association between PNI and mortality risk in patients with pressure ulcers was nonlinear, following an L shape for the prediction of 365-day and 180-day all-cause mortality. This finding suggested that the PNI can serve as an early clinical biomarker for stratifying high-risk patients and informing the development of personalized care strategies.

## Supporting information

S1 Table Missing number for risk variables and outcome variables. (DOCX)

S2 Table The baseline characteristics and outcomes between excluded and included participants. (DOCX)

S3 Table Variance inflation factor between variables. (DOCX)

S4 Table Association between clinical risk factors and mortality. (DOCX)

S5 Table Proportional hazards assumption tests for key variables in the multivariable cox models for each outcome. (DOCX)

S6 Table The association of the PNI with 365-day and 180-day mortality in male and female patients. (DOCX)

S7 Table Subgroup analysis of the effect of the PNI on 365-day all-cause mortality. (DOCX)

S8 Table Subgroup analysis of the effect of the PNI on 180-day all-cause mortality. (DOCX)

## Abbreviations

PNI: prognostic nutritional index
SOFA: Sequential organ failure assessment score
OASIS: Oxford acute severity of illness score
SBP: systolic blood pressure
DBP: diastolic blood pressure
SpO2: pulse blood oxygen saturation
PLT: platelets
BUN: blood urea nitrogen
PT: prothrombin time
LOS: length of stay
