# Supplementary material for: Association between prognostic nutritional index and long-term mortality in intensive care unit patients with pressure ulcers: A retrospective study
Source: PLoS One. 2026 Feb 10;21(2):e0341343. doi: 10.1371/journal.pone.0341343 (PMC12890147; doi:10.1371/journal.pone.0341343)
Supplement: S1 Table — (DOCX) [file pone.0341343.s001.docx]

Supplementary Table 1 missing number for risk variables and outcome variables

| Risk Variables | Missing number (%) |
| --- | --- |
| **Age, years** | 0 |
| **Male, n (%)** | 0 |
| **Weight, Kg** | 0 |
| **Smoking, n (%)** | 0 |
| **Race, n (%)** |  |
| White | 0 |
| Black | 0 |
| Asian | 0 |
| Others | 0 |
| **Severity of Illness** |  |
| SOFA | 1 (0.12%) |
| OASIS | 0 |
| **Vital Signs** |  |
| Temperature, ℃ | 12 (1.50%) |
| Heart rate, bpm | 4 (0.50%) |
| Respiratory rate, bpm | 4 (0.50%) |
| SBP, mmHg | 5 (0.62%) |
| DBP, mmHg | 5 (0.62%) |
| SpO2, % | 4 (0.50%) |
| **Commorbidities, n (%)** |  |
| Sepsis | 0 |
| Myocardial infarct | 0 |
| Heart failure | 0 |
| Chronic pulmonary disease | 0 |
| Cerebrovascular disease | 0 |
| Hypertension | 0 |
| Diabetes | 0 |
| Renal failure | 0 |
| **Treatments, n (%)** |  |
| Mechanical ventilation | 0 |
| Renal replacement therapy | 0 |
| **Laboratory parameters** |  |
| Lymphocyte, x 10^9/L | 0 |
| PLT, x 10^9/L | 0 |
| Hemoglobin, g/dL | 0 |
| Albumin, g/dL | 0 |
| Creatinine, mg/dL | 1 (0.12%) |
| BUN, mg/dL | 1 (0.12%) |
| PT, s | 30 (3.76%) |
| Glucose, mg/dL | 1 (0.12%) |

Abbreviations: SOFA, Sequential organ failure assessment score; OASIS, Oxford acute severity of illness score; SBP, systolic blood pressure; DBP, diastolic blood pressure; SpO2, pulse blood oxygen saturation; PLT, platelets; BUN, blood urea nitrogen; PT, prothrombin time.
