# Supplementary material for: Association between prognostic nutritional index and long-term mortality in intensive care unit patients with pressure ulcers: A retrospective study
Source: PLoS One. 2026 Feb 10;21(2):e0341343. doi: 10.1371/journal.pone.0341343 (PMC12890147; doi:10.1371/journal.pone.0341343)
Supplement: S2 Table — (DOCX) [file pone.0341343.s002.docx]

Supplementary Table 2 The baseline characteristics and outcomes between excluded and included participants.

| **Characteristics** | **Overall**  **n = 3251** | **Include**  **n = 796** | **Exclude**  **n = 2254** | **P** |
| --- | --- | --- | --- | --- |
| **Age, years** | 71.75 (61.45-81.58) | 70.33 (60.91-79.96) | 72.34 (61.73-82.28) | 0.003 |
| **Male, n (%)** | 176 (57.7) | 451 (56.7) | 1309 (58.0) | 0.487 |
| **Weight, Kg** | 73.8 (60.3-90.0) | 73.5 (60.9-90.9) | 73.9 (60.2-90.0) | 0.876 |
| **Smoking, n (%)** | 174 (5.7) | 40 (5.0) | 134 (5.9) | 0.336 |
| **Race, n (%)** |  |  |  | 0.047 |
| White | 2049 (67.1) | 509 (63.9) | 1540 (68.3) |  |
| Black | 414 (13.5) | 108 (13.6) | 306 (13.5) |  |
| Asian | 59 (1.9) | 20 (2.5) | 39 (1.7) |  |
| Others | 528 (17.3) | 159 (20.0) | 369 (16.3) |  |
| **Severity of Illness** |  |  |  |  |
| SOFA | 6 (4-9) | 7 (4-10) | 6 (4-8) | < 0.001 |
| OASIS | 34 (29-40) | 35 (29-42) | 34 (28-40) | < 0.001 |
| **Vital Signs** |  |  |  |  |
| Temperature, ℃ | 36.72 (36.39-37.11) | 36.72 (36.39-37.11) | 36.72 (36.39-37.11) | 0.827 |
| Heart rate, bpm | 91 (78-106) | 94 (80-110) | 91 (78-105) | < 0.001 |
| Respiratory rate, bpm | 20 (16-24) | 20 (16-25) | 20 (16-24) | 0.193 |
| SBP, mmHg | 117 (101-134) | 114 (100-132) | 117 (102-135) | 0.011 |
| DBP, mmHg | 64 (53-76) | 64 (53-76) | 64 (53-75) | 0.755 |
| SpO2, % | 98 (95-100) | 98 (95-100) | 98 (95-100) | 0.277 |
| **Commorbidities, n (%)** |  |  |  |  |
| Sepsis | 2214 (72.5) | 611 (76.8) | 1603 (71.1) | 0.002 |
| Myocardial infarct | 592 (19.4) | 154 (19.3) | 438 (19.4) | 0.958 |
| Heart failure | 1262 (41.3) | 324 (40.7) | 938 (41.6) | 0.654 |
| Chronic pulmonary disease | 853 (27.9) | 206 (25.9) | 647 (28.7) | 0.127 |
| Cerebrovascular disease | 546 (17.9) | 127 (16.0) | 419 (18.5) | 0.096 |
| Hypertension | 964 (31.6) | 228 (28.6) | 736 (32.6) | 0.036 |
| Diabetes | 1322 (43.3) | 341 (42.8) | 981 (43.5) | 0.738 |
| Renal failure | 1114 (36.5) | 306 (38.4) | 808 (35.8) | 0.191 |
| **Treatments, n (%)** |  |  |  |  |
| Mechanical ventilation | 1387 (45.4) | 383 (48.1) | 1004 (44.5) | 0.082 |
| Renal replacement therapy | 518 (16.9) | 166 (14.6) | 352 (15.6) | < 0.001 |
| **Laboratory parameters** |  |  |  |  |
| PLT, x 10^9/L | 224 (149-309) | 216 (132-305) | 225 (155-310) | 0.012 |
| Hemoglobin, g/dL | 9.6 (8.3-11.1) | 9.5 (8.0-11.0) | 9.600 (8.4, 11.2) | 0.049 |
| Creatinine, mg/dL | 1.2 (0.7-2.2) | 1.4 (0.8-2.6) | 1.2 (0.7-2.1) | < 0.001 |
| BUN, mg/dL | 30 (18-51) | 34 (21-56) | 29 (18-49) | < 0.001 |
| PT, s | 15.2 (13.3-19.1) | 15.3 (13.4-19.5) | 15.1 (13.3-18.9) | 0.254 |
| Glucose, mg/dL | 127 (101-168) | 129 (101-172) | 126 (101-166) | 0.526 |
| **Outcome** |  |  |  |  |
| LOS hospital, days | 13.0 (7.0-24.1) | 14.7 (7.6-27.2) | 12.5 (6.9-23.3) | < 0.001 |
| 180-day mortality, n (%) | 1470 (48.1) | 413 (51.9) | 1057 (46.8) | 0.015 |
| 365-day mortality, n (%) | 1750 (57.3) | 476 (59.8) | 1274 (56.5) | 0.108 |

Abbreviations: SOFA, Sequential organ failure assessment score; OASIS, Oxford acute severity of illness score; SBP, systolic blood pressure; DBP, diastolic blood pressure; SpO2, pulse blood oxygen saturation; PLT, platelets; BUN, blood urea nitrogen; PT, prothrombin time; LOS, length of stay.
