# Supplementary material for: Association between prognostic nutritional index and long-term mortality in intensive care unit patients with pressure ulcers: A retrospective study
Source: PLoS One. 2026 Feb 10;21(2):e0341343. doi: 10.1371/journal.pone.0341343 (PMC12890147; doi:10.1371/journal.pone.0341343)
Supplement: S3 Table — (DOCX) [file pone.0341343.s003.docx]

Supplementary Table 3 Variance inflation factor between variables

| **Variables** | **VIF** |
| --- | --- |
| Age | 1.497 |
| Male | 1.153 |
| Weight | 1.267 |
| Smoking | 1.054 |
| Race | 1.055 |
| Temperature | 1.173 |
| SBP | 1.510 |
| DBP | 1.611 |
| SpO2 | 1.102 |
| Sepsis | 1.250 |
| Myocardial infarct | 1.104 |
| Heart failure | 1.341 |
| Chronic pulmonary disease | 1.126 |
| Cerebrovascular disease | 1.075 |
| Hypertension | 1.497 |
| Diabetes | 1.172 |
| Renal failure | 1.715 |
| Renal replacement therapy | 1.475 |
| PNI | 1.015 |

Abbreviations: PNI, prognostic nutritional index; SBP, systolic blood pressure; DBP, diastolic blood pressure; SpO2, pulse blood oxygen saturation.
