# Supplementary material for: Association between prognostic nutritional index and long-term mortality in intensive care unit patients with pressure ulcers: A retrospective study
Source: PLoS One. 2026 Feb 10;21(2):e0341343. doi: 10.1371/journal.pone.0341343 (PMC12890147; doi:10.1371/journal.pone.0341343)
Supplement: S4 Table — (DOCX) [file pone.0341343.s004.docx]

Supplementary Table 4 Association between clinical risk factors and mortality.

| **Variables** | **Univariate analysis**  **HR (95% CI, 365 days)** | **P-value** | **Univariate analysis**  **HR (95% CI, 180 days)** | **P-value** |
| --- | --- | --- | --- | --- |
| Age | 1.036 (1.025-1.047) | < 0.001 | 1.034 (1.023-1.044) | < 0.001 |
| Male | 1.119 (0.841-1.489) | 0.439 | 1.001 (0.755-1.324) | 0.820 |
| Weight | 0.992 (0.986-0.997) | 0.003 | 0.991 (0.985-0.997) | 0.002 |
| Smoking | 1.009 (0.527-1.931) | 0.979 | 1.270 (0.668-2.415) | 0.467 |
| Race | 0.907 (0.806-1.021) | 0.105 | 0.954 (0.849-1.072) | 0.429 |
| Temperature | 0.714 (0.597-0.854) | < 0.001 | 0.699 (0.585-0.835) | < 0.001 |
| SBP | 0.995 (0.989-1.004) | 0.056 | 0.996 (0.990-1.001) | 0.135 |
| DBP | 0.997 (0.989-1.004) | 0.389 | 0.999 (0.992-1.006) | 0.751 |
| SpO2 | 0.993 (0.963-1.025) | 0.677 | 0.983 (0.954-1.014) | 0.286 |
| Sepsis | 1.760 (1.263-2.452) | 0.001 | 1.572 (1.128-2.190) | 0.007 |
| Myocardial infarct | 1.406 (0.972-2.033) | 0.071 | 1.697 (1.183-2.434) | 0.004 |
| Heart failure | 1.559 (1.163-2.090) | 0.003 | 1.652 (1.241-2.199) | 0.001 |
| Chronic pulmonary disease | 1.558 (1.115-2.175) | 0.009 | 1.306 (0.949-1.797) | 0.102 |
| Cerebrovascular disease | 1.380 (0.927-2.056) | 0.113 | 1.212 (0.828-1.776) | 0.323 |
| Hypertension | 0.614 (0.450-0.837) | 0.002 | 0.605 (0.444-0.826) | 0.002 |
| Diabetes | 1.268 (0.951-1.691) | 0.106 | 1.065 (0.804-1.411) | 0.659 |
| Renal failure | 2.282 (1.682-3.097) | < 0.001 | 1.918 (1.434-2.566) | < 0.001 |
| Renal replacement therapy | 1.623 (1.128-2.336) | 0.009 | 1.487 (1.051-2.104) | 0.025 |

Abbreviations: SBP, systolic blood pressure; DBP, diastolic blood pressure; SpO2, pulse blood oxygen saturation.
