# Supplementary material for: Association between prognostic nutritional index and long-term mortality in intensive care unit patients with pressure ulcers: A retrospective study
Source: PLoS One. 2026 Feb 10;21(2):e0341343. doi: 10.1371/journal.pone.0341343 (PMC12890147; doi:10.1371/journal.pone.0341343)
Supplement: S5 Table — (DOCX) [file pone.0341343.s005.docx]

Supplementary Table 5 Proportional hazards assumption tests for key variables in the multivariable cox models for each outcome.

| **Variables** | **Outcomes** | **P value** |
| --- | --- | --- |
| Age | 365-day mortality | 0.079 |
|  | 180-day mortality | 0.069 |
| Male | 365-day mortality | 0.216 |
|  | 180-day mortality | 0.444 |
| Weight | 365-day mortality | 0.932 |
|  | 180-day mortality | 0.646 |
| Smoking | 365-day mortality | 0.586 |
|  | 180-day mortality | 0.839 |
| Race | 365-day mortality | 0.296 |
|  | 180-day mortality | 0.537 |
| Temperature | 365-day mortality | 0.441 |
|  | 180-day mortality | 0.558 |
| SBP | 365-day mortality | 0.993 |
|  | 180-day mortality | 0.925 |
| DBP | 365-day mortality | 0.555 |
|  | 180-day mortality | 0.457 |
| SpO2 | 365-day mortality | 0.203 |
|  | 180-day mortality | 0.408 |
| Sepsis | 365-day mortality | 0.126 |
|  | 180-day mortality | 0.055 |
| Myocardial infarct | 365-day mortality | 0.601 |
|  | 180-day mortality | 0.931 |
| Heart failure | 365-day mortality | 0.879 |
|  | 180-day mortality | 0.670 |
| Chronic pulmonary disease | 365-day mortality | 0.483 |
|  | 180-day mortality | 0.815 |
| Cerebrovascular disease | 365-day mortality | 0.211 |
|  | 180-day mortality | 0.327 |
| Hypertension | 365-day mortality | 0.371 |
|  | 180-day mortality | 0.344 |
| Diabetes | 365-day mortality | 0.365 |
|  | 180-day mortality | 0.836 |
| Renal failure | 365-day mortality | 0.849 |
|  | 180-day mortality | 0.714 |
| Renal replacement therapy | 365-day mortality | 0.327 |
|  | 180-day mortality | 0.204 |
| PNI | 365-day mortality | 0.633 |
|  | 180-day mortality | 0.533 |

Abbreviations: SBP, systolic blood pressure; DBP, diastolic blood pressure; SpO2, pulse blood oxygen saturation.
