# Supplementary material for: Association between prognostic nutritional index and long-term mortality in intensive care unit patients with pressure ulcers: A retrospective study
Source: PLoS One. 2026 Feb 10;21(2):e0341343. doi: 10.1371/journal.pone.0341343 (PMC12890147; doi:10.1371/journal.pone.0341343)
Supplement: S6 Table — (DOCX) [file pone.0341343.s006.docx]

Supplementary Table 6 The association of the PNI with 365-day and 180-day mortality in male and female patients

| **Variables** | **Model 1** | | **Model 2** | | **Model 3** | |
| --- | --- | --- | --- | --- | --- | --- |
|  | **HR (95% CI)** | **p-value** | **HR (95% CI)** | **p-value** | **HR (95% CI)** | **p-value** |
| **365-day mortality** |  |  |  |  |  |  |
| **Male** |  |  |  |  |  |  |
| Quartile 1 group | ref |  | ref |  | ref |  |
| Quartile 2 group | 0.853 (0.616-1.180) | 0.336 | 0.813 (0.586-1.126) | 0.213 | 0.902 (0.645-1.260) | 0.545 |
| Quartile 3 group | 0.793 (0.570-1.104) | 0.169 | 0.732 (0.525-1.021) | 0.066 | 0.765 (0.540-1.084) | 0.132 |
| Quartile 4 group | 0.597 (0.424-0.841) | 0.003 | 0.591 (0.417-0.837) | 0.003 | 0.637 (0.439-0.923) | 0.017 |
| **Female** |  |  |  |  |  |  |
| Quartile 1 group | ref |  | ref |  | ref |  |
| Quartile 2 group | 0.665 (0.458-0.965) | 0.032 | 0.605 (0.415-0.882) | 0.009 | 0.666 (0.453-0.980) | 0.039 |
| Quartile 3 group | 0.585 (0.401-0.856) | 0.006 | 0.557 (0.379-0.820) | 0.003 | 0.638 (0.430-0.947) | 0.026 |
| Quartile 4 group | 0.540 (0.366-0.796) | 0.002 | 0.489 (0.329-0.728) | < 0.001 | 0.624 (0.406-0.961) | 0.032 |
| **180-day mortality** |  |  |  |  |  |  |
| **Male** |  |  |  |  |  |  |
| Quartile 1 group | ref |  | ref |  | ref |  |
| Quartile 2 group | 0.816 (0.573-1.162) | 0.260 | 0.789 (0.553-1.125) | 0.190 | 0.898 (0.623-1.293) | 0.562 |
| Quartile 3 group | 0.860 (0.606-1.219) | 0.397 | 0.809 (0.569-1.150) | 0.238 | 0.843 (0.583-1.220) | 0.365 |
| Quartile 4 group | 0.561 (0.384-0.819) | 0.003 | 0.567 (0.386-0.833) | 0.004 | 0.640 (0.425-0.963) | 0.032 |
| **Female** |  |  |  |  |  |  |
| Quartile 1 group | ref |  | ref |  | ref |  |
| Quartile 2 group | 0.672 (0.458-0.987) | 0.043 | 0.610 (0.413-0.901) | 0.013 | 0.663 (0.445-0.989) | 0.044 |
| Quartile 3 group | 0.554 (0.371-0.828) | 0.004 | 0.527 (0.351-0.793) | 0.002 | 0.607 (0.399-0.923) | 0.019 |
| Quartile 4 group | 0.465 (0.305-0.711) | < 0.001 | 0.421 (0.273-0.650) | < 0.001 | 0.537 (0.336-0.857) | 0.009 |

Model 1 was unadjusted

Model 2 was adjusted for age, gender, and race.

Model 3 was adjusted for the variables in model 2 and further adjusted for weight, smoking, temperature, SBP, DBP, SpO2, sepsis, myocardial infarction, heart failure, chronic pulmonary disease, cerebrovascular disease, hypertension, diabetes, renal failure, and renal replacement therapy.
