# Supplementary material for: Association between prognostic nutritional index and long-term mortality in intensive care unit patients with pressure ulcers: A retrospective study
Source: PLoS One. 2026 Feb 10;21(2):e0341343. doi: 10.1371/journal.pone.0341343 (PMC12890147; doi:10.1371/journal.pone.0341343)
Supplement: S7 Table — (DOCX) [file pone.0341343.s007.docx]

Supplementary Table 7 Subgroup analysis of the effect of the PNI on 365-day all-cause mortality

|  | **G1** | **G2** | **G3** | **G4** | **P for interaction** |
| --- | --- | --- | --- | --- | --- |
| **Age** |  |  |  |  | 0.400 |
| > 65 | Ref | 0.669 (0.502-0.890)** | 0.611 (0.459-0.812)** | 0.515 (0.382-0.695)*** |  |
| ≤ 65 | Ref | 0.999 (0.625-1.596) | 0.816 (0.493-1.353) | 0.681 (0.413-1.123) |  |
| **Gender** |  |  |  |  | 0.504 |
| Male | Ref | 0.853 (0.616-1.180) | 0.793 (0.570-1.104) | 0.597 (0.424-0.841)** |  |
| Female | Ref | 0.665 (0.458-0.965)* | 0.585 (0.401-0.856)** | 0.540 (0.366-0.796)** |  |
| **Sepsis** |  |  |  |  | 0.246 |
| Yes | Ref | 0.769 (0.589-1.005) | 0.727 (0.552-0.958)* | 0.646 (0.484-0.861)** |  |
| No | Ref | 0.729 (0.402-1.320) | 0.605 (0.341-1.072) | 0.440 (0.249-0.776)** |  |
| **Hypertension** |  |  |  |  | 0.679 |
| Yes | Ref | 0.622 (0.367-1.053) | 0.598 (0.363-0.986)* | 0.530 (0.324-0.865)* |  |
| No | Ref | 0.811 (0.616-1.068) | 0.741 (0.556-0.986)* | 0.599 (0.443-0.810)** |  |
| **Diabetes** |  |  |  |  | 0.968 |
| Yes | Ref | 0.810 (0.558-1.177) | 0.577 (0.390-0.852)** | 0.590 (0.402-0.866)** |  |
| No | Ref | 0.710 (0.513-0.984)* | 0.780 (0.565-1.077) | 0.525 (0.369-0.747)*** |  |
| **Myocardial infarct** |  |  |  |  | 0.934 |
| Yes | Ref | 0.682 (0.397-1.173) | 0.529 (0.312-0.895)* | 0.637 (0.365-1.109) |  |
| No | Ref | 0.788 (0.600-1.035) | 0.733 (0.553-0.972)* | 0.555 (0.416-0.741)*** |  |
| **Heart failure** |  |  |  |  | 0.211 |
| Yes | Ref | 0.721 (0.496-1.046) | 0.678 (0.468-0.983)* | 0.445 (0.299-0.662)*** |  |
| No | Ref | 0.755 (0.546-1.043) | 0.648 (0.462-0.909)* | 0.640 (0.457-0.896)** |  |
| **Chronic pulmonary disease** |  |  |  |  | 0.725 |
| Yes | Ref | 1.244 (0.789-1.961) | 0.617 (0.375-1.015) | 0.605 (0.379-0.963)* |  |
| No | Ref | 0.653 (0.489-0.873)** | 0.719 (0.540-0.958)* | 0.545 (0.400-0.742)*** |  |
| **Cerebrovascular disease** |  |  |  |  | 0.564 |
| Yes | Ref | 0.784 (0.423-1.454) | 0.594 (0.302-1.166) | 0.652 (0.358-1.186) |  |
| No | Ref | 0.751 (0.576-0.980)* | 0.704 (0.539-0.919)* | 0.530 (0.397-0.708)*** |  |

* p< 0.05, ** p< 0.01, *** p< 0.001.
