# Supplementary material for: Association between prognostic nutritional index and long-term mortality in intensive care unit patients with pressure ulcers: A retrospective study
Source: PLoS One. 2026 Feb 10;21(2):e0341343. doi: 10.1371/journal.pone.0341343 (PMC12890147; doi:10.1371/journal.pone.0341343)
Supplement: S8 Table — (DOCX) [file pone.0341343.s008.docx]

Supplementary Table 8 Subgroup analysis of the effect of the PNI on 180-day all-cause mortality

|  | **G1** | **G2** | **G3** | **G4** | **P for interaction** |
| --- | --- | --- | --- | --- | --- |
| **Age** |  |  |  |  | 0.851 |
| > 65 | Ref | 0.643 (0.473-0.873)** | 0.618 (0.457-0.835)** | 0.499 (0.361-0.688)*** |  |
| ≤ 65 | Ref | 1.024 (0.624-1.678) | 0.894 (0.529-1.512) | 0.507 (0.282-0.909)* |  |
| **Gender** |  |  |  |  | 0.264 |
| Male | Ref | 0.816 (0.573-1.162) | 0.860 (0.606-1.219) | 0.561 (0.384-0.819)** |  |
| Female | Ref | 0.672 (0.458-0.987)* | 0.554 (0.371-0.828)** | 0.465 (0.305-0.711)*** |  |
| **Sepsis** |  |  |  |  | 0.195 |
| Yes | Ref | 0.761 (0.572-1.012) | 0.740 (0.552-0.991)* | 0.594 (0.433-0.815)** |  |
| No | Ref | 0.653 (0.349-1.222) | 0.619 (0.345-1.111) | 0.357 (0.193-0.663)** |  |
| **Hypertension** |  |  |  |  | 0.416 |
| Yes | Ref | 0.504 (0.282-0.901)* | 0.578 (0.343-0.973)* | 0.427 (0.249-0.733)** |  |
| No | Ref | 0.825 (0.616-1.104) | 0.771 (0.570-1.043) | 0.562 (0.404-0.782)** |  |
| **Diabetes** |  |  |  |  | 0.627 |
| Yes | Ref | 0.696 (0.465-1.041) | 0.579 (0.385-0.872)** | 0.490 (0.321-0.748)** |  |
| No | Ref | 0.760 (0.541-1.069) | 0.807 (0.574-1.135) | 0.509 (0.347-0.746)** |  |
| **Myocardial infarct** |  |  |  |  | 0.870 |
| Yes | Ref | 0.686 (0.395-1.192) | 0.494 (0.286-0.853)* | 0.640 (0.363-1.128) |  |
| No | Ref | 0.759 (0.566-1.018) | 0.770 (0.571-1.036) | 0.481 (0.348-0.666)*** |  |
| **Heart failure** |  |  |  |  | 0.285 |
| Yes | Ref | 0.681 (0.463-1.000) | 0.622 (0.422-0.916)* | 0.393 (0.257-0.600)*** |  |
| No | Ref | 0.741 (0.521-1.055) | 0.723 (0.506-1.032) | 0.582 (0.399-0.849)** |  |
| **Chronic pulmonary disease** |  |  |  |  | 0.767 |
| Yes | Ref | 1.407 (0.863-2.295) | 0.674 (0.391-1.163) | 0.583 (0.343-0.989)* |  |
| No | Ref | 0.590 (0.432-0.805)** | 0.721 (0.535-0.971)* | 0.485 (0.347-0.679)*** |  |
| **Cerebrovascular disease** |  |  |  |  | 0.686 |
| Yes | Ref | 0.683 (0.354-1.315) | 0.529 (0.257-1.092) | 0.578 (0.306-1.091) |  |
| No | Ref | 0.746 (0.562-0.990)* | 0.736 (0.556-0.974)* | 0.476 (0.345-0.655)*** |  |

* p< 0.05, ** p< 0.01, *** p< 0.001.
